# Thymosin Alpha‐1 Provides Direct Neuroprotection by Engaging the Orexin Receptor HCRTR1 to Suppress Neuronal Necroptosis

**DOI:** 10.1002/advs.202522372

**Published:** 2026-09-03

**Authors:** Xinmei Kang, Shisi Wang, Liubin Zhang, Mengyan Hu, Xiaotao Su, Yuxin Liu, Qin Qin, Haotong Yi, Liling Yuan, Wei Cai, Lei Wei, Zhengqi Lu

**Affiliations:** ^1^ Department of Neurology Mental and Neurological Disease Research Center the Third Affiliated Hospital of Sun Yat‐Sen University Guangzhou China; ^2^ Guangdong Provincial Key Laboratory of Brain Function and Disease Guangzhou China

**Keywords:** hypocretin (Orexin) receptor 1, necroptosis, thymosin alpha‐1

## Abstract

Communication between the immune system and the brain is critical for neuronal health, yet the molecular signals that mediate this crosstalk are not fully understood. Here, we uncover an unexpected and direct neuroprotective axis linking the thymus to the central nervous system (CNS). We show that the thymus‐derived peptide Thymosin alpha‐1 (Tα1) functions as a non‐canonical ligand for the Hypocretin (Orexin) Receptor 1 (HCRTR1), a classical neuropeptide receptor on neurons. This engagement shields neurons from cell death by suppressing the activity of Receptor‐Interacting Protein Kinase 3 (RIPK3), a central executioner of necroptosis. The physiological relevance of this pathway is highlighted in ischemic stroke, where we found that circulating Tα1 levels were significantly reduced in patients and mice, strongly correlating with disease severity. Genetic deletion of the Tα1‐encoding gene *Ptma* exacerbated stroke injury, whereas therapeutic administration of Tα1 conferred robust neuroprotection and improved functional recovery. Our findings identify a novel thymus‐brain signaling pathway, revealing a new neuroprotective function for an immune peptide and an unexpected role for a canonical neuronal receptor. This work establishes Tα1 as a promising dual‐action therapeutic candidate, capable of both direct neuronal protection and systemic immunomodulation.

## Graphical abstract

In this study, we unveil a thymus‐brain endocrine axis wherein Tα1 engages HCRTR1 to inhibit RIPK3‐mediated necroptosis. This study nominates the Tal‐HCRTR1‐RIPK3 axis as a potent and clinically translatable therapeutic target for stroke and neurodegeneration.

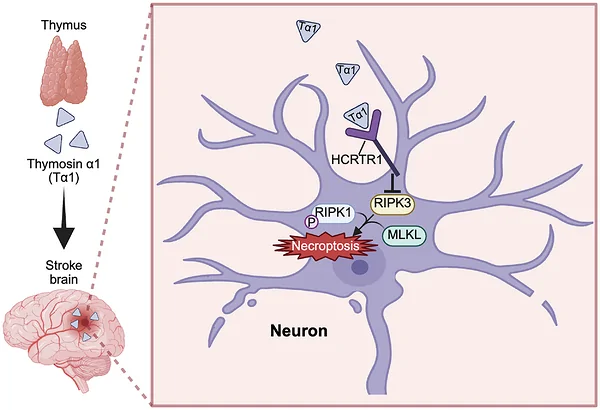

## 1 Introduction

The nervous and immune systems engage in a continuous, intricate dialogue that is fundamental to maintaining homeostasis and responding to injury [1, 2].While it is well‐established that peripheral immune signals can profoundly influence brain function, this communication is often viewed as indirect, mediated by inflammatory cascades or the infiltration of immune cells [3, 4]. The extent to which signaling molecules from peripheral immune organs can cross the blood‐brain barrier (BBB) to directly engage neuronal receptors and trigger specific intracellular pathways remains a key unanswered question in neurobiology. Uncovering such direct, non‐canonical signaling axes would represent a significant advance in our understanding of neuro‐immune communication.

A central organ in orchestrating systemic immunity is the thymus, which processes Prothymosin alpha (PTMA) to secrete its most active peptide, Thymosin alpha‐1 (Tα1) [5, 6] Interestingly, PTMA is also expressed endogenously by neurons, where it is thought to play intracellular roles in proliferation and chromatin organization [7, 8]. While the administration of exogenous Tα1 has been shown to be neuroprotective in various models of brain injury, these effects have often been attributed to broad anti‐inflammatory or anti‐oxidative actions [9, 10, 11]. Additionally, Tα1 has been reported to antagonize dexamethasone (DEX)‐induced apoptosis of CD4^+^CD8^+^ thymocytes, suggesting a role in modulating cell survival decisions beyond classical immune modulation [12]. However, whether Tα1 can function as an extracellular signaling molecule to directly regulate neuronal fate through a receptor‐mediated mechanism, which bypassing its classical immunomodulatory role remains entirely unknown. The absence of any known cell surface receptor for Tα1 on neurons has precluded mechanistic investigation into such direct, non‐immune cytoprotection, leaving a critical gap in our understanding of how this peptide exerts its protective effects in the injured brain. We therefore hypothesized that Tα1, acting as a hormone‐like signal, engages a specific neuronal receptor to initiate a direct, pro‐survival intracellular cascade.

Here, we identify and characterize an unexpected and potent thymus‐neuron protective axis. We show that Tα1 crosses the BBB to act as a novel, non‐canonical ligand for the Hypocretin (Orexin) Receptor 1 (HCRTR1), a classical G protein‐coupled receptor integral to arousal and metabolic regulation [13]. This engagement triggers the suppression of Receptor‐Interacting Protein Kinase 3 (RIPK3), a key driver of necroptosis, a lytic and pro‐inflammatory form of programmed cell death [14, 15]. We demonstrate the profound physiological relevance of this pathway in the context of ischemic stroke, a condition where neuronal loss dictates functional outcome [16]. We found that Tα1 is down‐regulated after stroke and that its therapeutic administration confers robust neuroprotection. Our discovery of a thymic peptide co‐opting a classical neuropeptide receptor to control a specific neuronal death pathway fundamentally redefines the biological role of Tα1 and presents the Tα1‐HCRTR1 axis as a powerful and previously unknown mechanism of cerebral defense.

## 2 Materials and Methods

### 2.1 Study Approval

The clinical studies were approved by the Medical Ethics Committee of the Third Affiliated Hospital of Sun Yat‐Sen University (Permit ID: RG2025‐012‐02). All participants had been given the informed consent according to the principles illustrated in Declaration of Helsinki. All Mice were used in accordance with the Guide for Care and Use of Laboratory Animals of the National Institute of Health, and protocols were approved by the Institutional Animal Care and Use Committee of The Third Affiliated Hospital of Sun Yat‐sen University (Permit ID: K2024‐01‐125).

### 2.2 Human Material

15 healthy donors and consecutive 28 AIS patients within 7 days of symptom onset were prospectively enrolled in the Third Affiliated Hospital of Sun Yat‐sen University from July 2024 to December 2025. Blood samples were collected at the time of admission, prior to any therapeutic intervention. Stroke severity was assessed by certified neurologists using the National Institutes of Health Stroke Scale (NIHSS). Infarct volumes were quantified on diffusion‐weighted MRI using the ABC/2 method. Systemic inflammation indices, including white blood cell count and C‐reactive protein were obtained from routine clinical laboratory tests. Demographic characteristics of the research cohort are summarized in Table S1.

### 2.3 Animals

8‐12‐weeks‐old, male C57/Bl6 wild‐type mice were purchased from Guangdong Medical Laboratory Animal Center (Guangzhou, China). All animal experimental procedures were approved by the Medical Ethics Committee of the Third Affiliated Hospital of Sun Yat‐sen University. Mice were group‐housed a 12:12 light/dark cycle, temperature with 24°C±2°C, humidity between 30%–70%, with ad libitum access to food and water. Animals were randomly assigned to different groups. To ensure the induction of ischemic stroke and eliminate the protection of estrogen, male mice that were 8–12 weeks old and with weight of 20–25 g were included in the in vivo experiments.

### 2.4 Generation of Ptma^+/−^ Mouse

The PTMA heterozygous knockout mouse (*Ptma* ^+/−^) was originally generated by the International Knockout Mouse Consortium(KOMP; MGl:5548964). Consistent with previous reports, Homozygous *Ptma* ^−/−^ is embryonic lethal [8]. The *Ptma* ^+/−^ mice were used in this study and purchased from GemPharmatech (Guangzhou, China). According to the structure of *Ptma* gene, exon2 of exon5 *Ptma*‐201(ENSMUST00000045897.14) transcript is recommended as the knockout region. The brief process is as follows: gRNA was transcribed in vitro, donor was constructed Cas9, gRNA and donor were micro‐injected into the fertilized eggs of C57BL/6JGpt mice. Fertilized eggs were transplanted to obtain positive F0 mice which were confirmed by PCR and sequencing. A stable F1 generation mouse model was obtained by mating positive F0 generation mice with C57BL/6JGpt mice.

### 2.5 Surface Plasmon Resonance (SPR)

Recombinant HCRTR1 were coupled onto a CM5 chip (#BR‐1005‐30) via carboxyl groups on the dextran. After incubation, a series concentration of Tα1 flowed through the protein‐CM5 system. The binding was tested and analyzed using Biacore T200 instrument.

### 2.6 Molecular Docking

The molecular interaction of Tα1, HCRTR1, RIPK3, RIPK1 and MLKL were ensured using methods established in the literature. The available crystal structures of the protein were downloaded from the Uniprot (https://www.uniprot.org). The three proteins were Tα1 (PDB ID: 2L9I), HCRTR1 (PDB ID: 4ZJ8), RIPK3 (PDB ID: 7MX3), and RIPK1 (PDB ID: 7JW7), MLKL (PDB ID: 7XMK). The files were saved in pdb format. Cluspro 2.0 docking server (https://cluspro.bu.edu/) was used to dock the interaction between Tα1 and HCRTR1 or HCRTR1 and RIPK3, RIPK1and MLKL. After the docking prediction by Cluspro, the interaction was saved in pdb format and the PyMOL software (http://www.pymol.org/pymol) was used to create the 3D structures. Meanwhile, weighted score was obtained from Cluspro.

### 2.7 Statistic Analysis

All analysis was performed using GraphPad Prism software (version 10.0). Values were shown as the mean ± standard deviation (SD) for data that were normally distributed. Student's *t* test: Unpaired parametric *t* test (two‐tailed) was performed in data comparison of two groups. Error bar represents standard error of mean ± standard deviation (SD). One‐way *ANOVA*: No matching *ANOVA* was performed in data comparison of three groups or more. Result was corrected for multiple comparisons using statistical hypothesis testing (Dunnett). Two‐way *ANOVA*: Two‐way *ANOVA* was performed in data on a quantitative dependent variableat multiple levels of two categorical independent variables. Results were considered significant at *p* < 0.05.

## 3 Results

### 3.1 Circulating Thymosin α1 is Down‐regulated After Acute Brain Injury and Correlates With Neurological Damage

**FIGURE 1:**
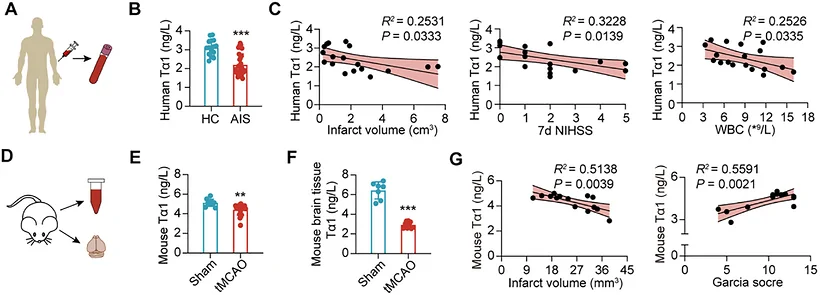
Circulating Thymosin α1 is down‐regulated after acute brain injury and correlates with neurological damage. (A–C) Healthy control (HC) (*N* = 15) and AIS patients (*N* = 28) peripheral blood serum and clinical data were collected and subjected to further analysis. (A) Diagram of peripheral blood test for patients. (B) Tα1 were detected by ELISA. ^***^ *p* < 0.001 compared to HC; by Student's *t* test (mean ± standard deviation). (C) The association between Tα1 with infarct volume, 7d NIHSS and WBC counts was evaluated with simple linear correlation analysis. (D) Diagram of peripheral blood and brain tissue test for mouse. (E) Tα1 in mouse peripheral blood were detected by ELISA. ^**^ *p* < 0.01 compared to Sham; by Student's *t* test (mean ± standard deviation). (F) Tα1 in mouse brain tissue were detected by ELISA. ^***^ *p* < 0.001 compared to Sham; by Student's *t* test (mean ± standard deviation). (G) The association between Tα1 with infarct volume and Garcia score was evaluated with simple linear correlation analysis.

To determine whether thymic peptides are involved in the response to acute brain injury, we first measured their circulating levels in a cohort of acute ischemic stroke (AIS) patients. This analysis revealed a significant and specific reduction in Tα1 levels compared to healthy controls (HC) (Figure 1A,B), whereas the related peptides Thymosin beta‐4 (Tβ4) and Thymosin beta‐10 (Tβ10) remained unchanged (Figure S1A,B). The reduction in Tα1 was clinically significant; lower circulating Tα1 levels correlated with larger infarct volumes, more severe neurological deficits, and heightened systemic inflammation (Figure 1C) and (Figure S1C,D). This specific peripheral depletion of Tα1 was recapitulated in a transient middle cerebral artery occlusion (tMCAO) mouse model of stroke, where Tβ4 and Tβ10 levels also remained unaffected (Figure 1D,E and Figure S1E,F). We next examined thymosin levels within the brain parenchyma itself. Interestingly, we found that the levels of all three peptides (Tα1, Tβ4, and Tβ1) were reduced in the ischemic hemisphere post‐stroke (Figure 1F and Figure S1G,H). Despite this general downregulation of thymosins within the brain, only Tα1 levels were functionally linked to stroke outcome. Critically, lower Tα1 concentrations in the brain tissue directly corresponded to larger infarct volumes and worse neurological scores (Figure 1G). The consistent and specific association between Tα1 depletion and the severity of brain injury in both humans and mice suggested that Tα1 plays an active role in the neuronal response to ischemic damage, prompting us to investigate its causal role in neuroprotection.

### 3.2 Endogenous Tα1 is Essential for Neuronal Survival and Functional Recovery After Stroke

**FIGURE 2:**
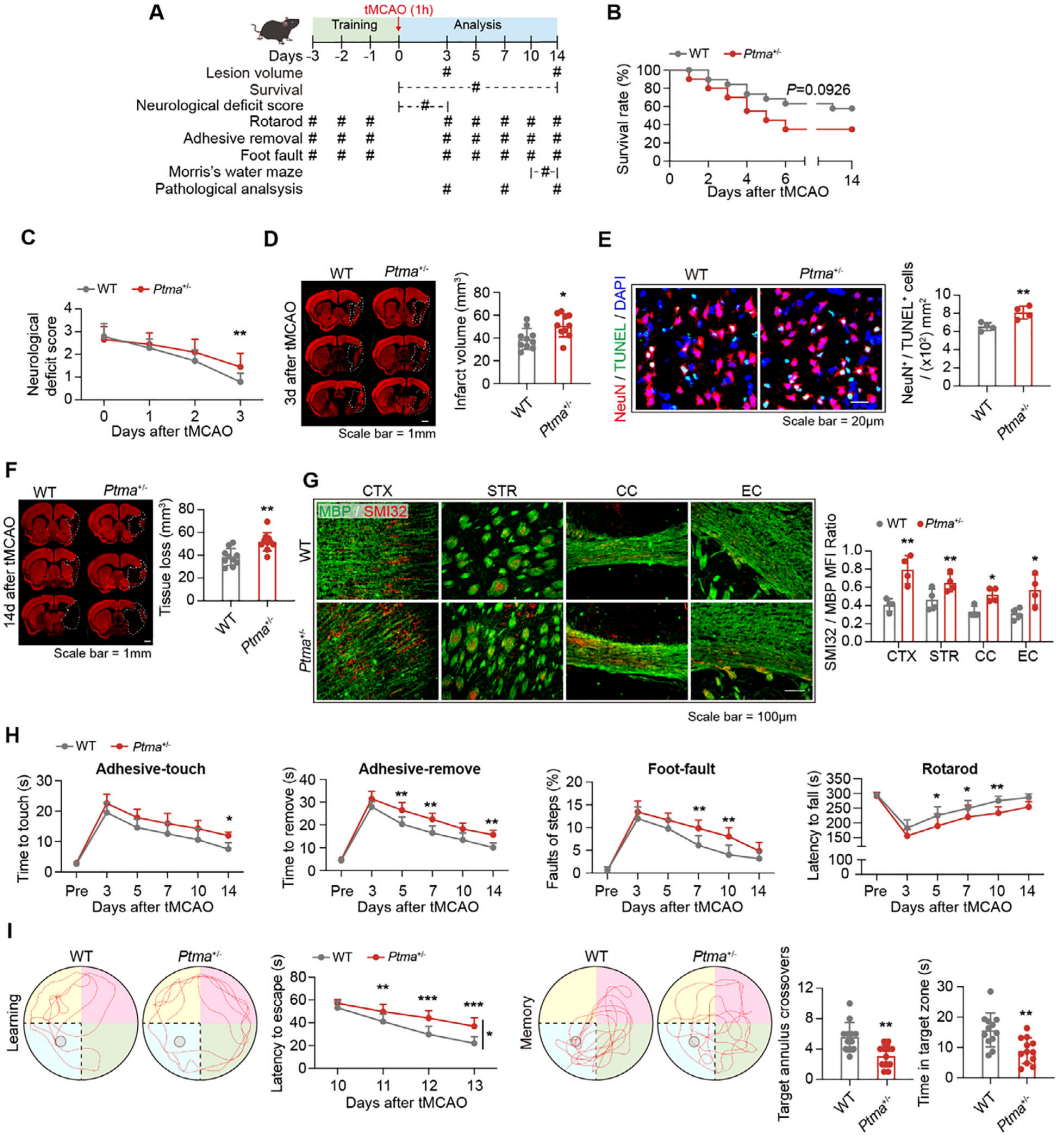
Endogenous Tα1 is essential for neuronal survival and functional recovery after stroke. WT and *Ptma^+/−^* male C57/Bl6 mice were subjected to 60 min of tMCAO and sacrificed at 3d or 14d after tMCAO. (A) Timeline of experimental design. (B) Survival of mice in each group was recorded at 0–14 days after tMCAO. *N* = 20 in each group. Exact *p* values are provided. compared with WT, by *log‐rank* test. (C) Neurological deficit score was assessed at 0–3d after tMCAO. *N* = 10 in each group. compared with WT, ^**^ *p* < 0.01, by Two‐way *ANOVA* (mean ± standard deviation). (D) Infarct volume was quantified with immunostaining of NeuN with coronal sections collected at 3d after tMCAO. Representative images were displayed. Dashed lines outlined the infarct area. *N* = 10 in each group.^*^ *p* < 0.05, compared with WT, by Student's *t* test (mean ± standard deviation). (E) Neuronal injury in stroke penumbra was evaluated with co‐immunostaining of NeuN (red) and TUNEL (green) at 3d after tMCAO. Representative images of NeuN^+^TUNEL^+^ neurons in stroke were displayed. *N* = 4 in each group. ^**^ *p* < 0.01, compared with WT, by Student's *t* test (mean ± standard deviation). (F) Infarct volume was quantified with immunostaining of NeuN with coronal sections collected at 14d after tMCAO. Representative images were displayed. Dashed lines outlined the infarct area. *N* = 10 in each group.^**^ *p* < 0.01, compared with WT, by Student's *t* test (mean ± standard deviation). (G) Representative images and quantification of white matter damage in the corpus callosum (CC), external capsule (EC), striatum (STR), and cortex (CTX) at 14 days post‐tMCAO. *N* = 4 in each group. ^*^ *p* < 0.05, ^**^ *p* < 0.01, compared with WT, by Two‐way *ANOVA* (mean ± standard deviation). (H) Sensorimotor functions of the stroke models were evaluated by adhesive‐removal test, foot‐fault test and rotarod test at 3–14d after tMCAO. *N* = 12 in each group, ^*^ *p* < 0.05, ^**^ *p* < 0.01, compared with WT, by Two‐way *ANOVA* (mean ± standard error). (I) Morris water maze test at 10–14 days after tMCAO. Representative images of the swim paths of mice in each group while the platform was present (learning phase) or removed (memory phase). *N* = 12 per group. ^**^ *p* < 0.01, ^***^ *p* < 0.001. In the learning phase, Two‐way *ANOVA* was used. In the memory phase, Student's *t* test and nonparametric test were used.

To directly test the hypothesis that Tα1 is neuroprotective, we subjected mice with a heterozygous knockout of the Tα1‐encoding gene *Ptma* (*Ptma* ^+/−^) to tMCAO (Figure 2A). The genetic deficiency of Tα1 led to a dramatic worsening of stroke outcomes. *Ptma* ^+/−^ mice exhibited lower survival rate compared to their wild‐type (WT) littermates (Figure 2B). In the acute phase, these mice displayed more severe neurological deficits (Figure 2C) and substantially larger infarct volumes (Figure 2D), which was associated with a marked increase in neuronal death in the peri‐infarct region (Figure 2E). This acute pathology translated into severe chronic damage, including greater tissue loss (Figure 2F), more extensive white matter injury (Figure 2G), and a heightened neuroinflammatory state characterized by the upregulation of multiple pro‐inflammatory factors (Figure S2A–C). Consistent with this widespread structural damage, *Ptma* ^+/−^ mice suffered from profound and lasting functional impairments, including significant deficits in sensorimotor tasks and cognitive impairments in learning and memory (Figure 2H,I). These loss‐of‐function data unequivocally establish that endogenous Tα1 is critical for mitigating ischemic brain injury and preserving neurological function, providing a strong rationale to test whether supplementing Tα1 could be a viable therapeutic strategy.

### 3.3 Exogenous Tα1 Administration Confers Robust Neuroprotection and Promotes Neuronal Survival

**FIGURE 3:**
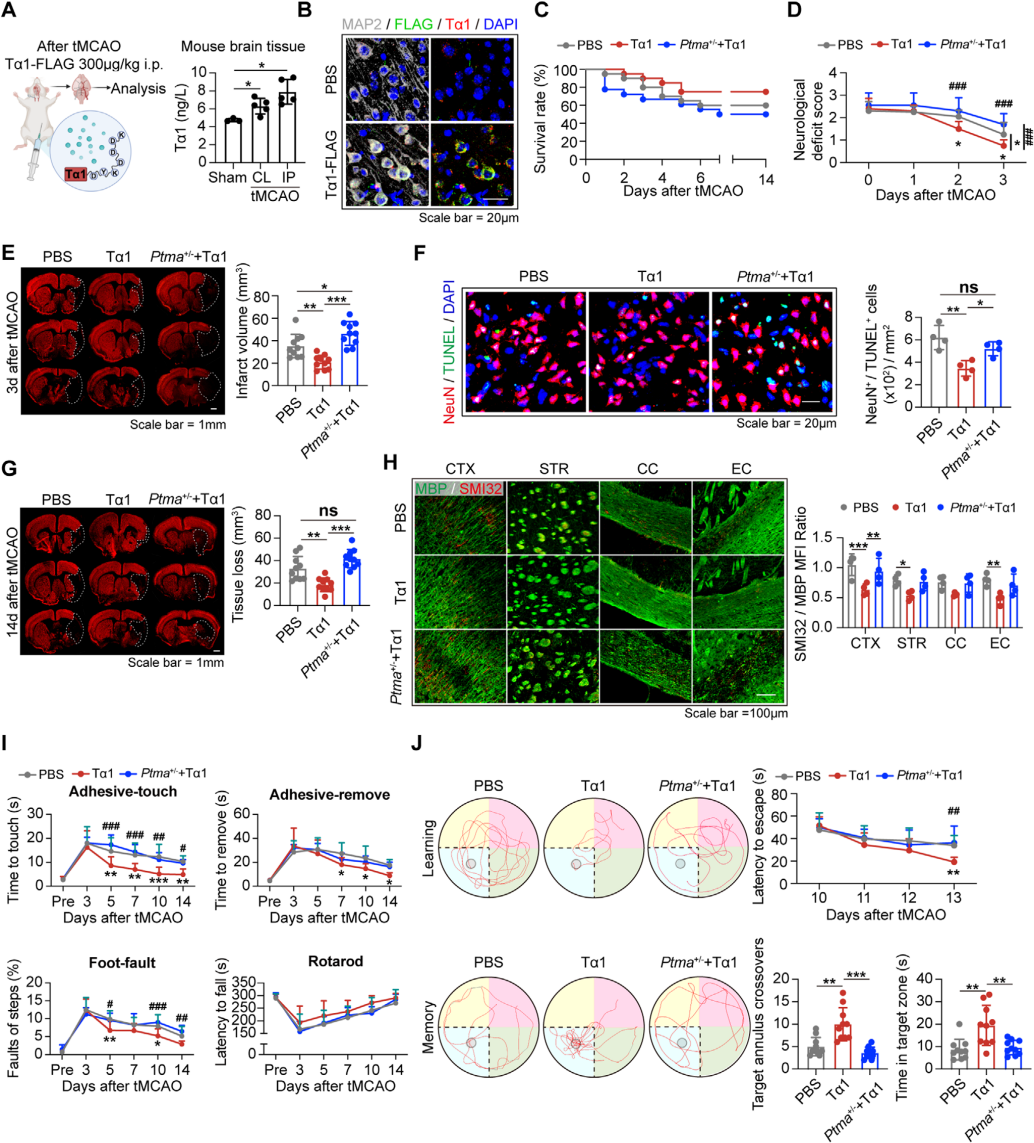
Exogenous Tα1 administration confers robust neuroprotection and promotes neuronal survival (A,B) Tα1 was conjugated with FLAG and administered intraperitoneally (i.p.) immediately following tMCAO. Mice were euthanized 24 h post‐administration, and brain tissues were harvested. (A) Tα1 in brain tissue was detected with ELISA. CL (Contralateral, non‐ischemic hemispheres), IP (Ipsilateral, ischemic hemispheres). *N* = 3 in Sham, *N* = 4 in CL and IP group. ^*^ *p* < 0.05, compared with by Sham, by One‐way *ANOVA* (mean ± standard deviation). (B) Brain tissue slices were conducted co‐immunostaining of MAP2/FLAG/Tα1 in ischemic penumbra. Representative images are shown. (C–J) WT and *Ptma^+/−^* male C5/Bl6 mice were subjected to 60 min of tMCAO and initiated daily intraperitoneal administration (i.p.) of either Tα1 (300 µg/kg) or PBS immediately after surgery for 14 days, mouse were sacrificed at 3d or 14d after tMCAO. (C) Survival of mice in each group was recorded at 0–14 days after tMCAO. *N* = 20 in PBS and Tα1group, *N* = 18 in *Ptma* ^+/^^−^ +Tα1 group. (D) Neurological deficit score was assessed at 0–3d after tMCAO. *N* = 10 in each group. ^*^ *p* < 0.05, ^**^ *p* < 0.01, Tα1 group vs. PBS group, by Two‐way *ANOVA* (mean ± standard deviation). **^###^** *P*< 0.001, Tα1 group vs. *Ptma^+/−^* +Tα1 group, by Two‐way *ANOVA* (mean ± standard deviation). (E) Infarct volume of stroke mice was quantified with immunostaining of NeuN with coronal sections collected at 3d post‐tMCAO. Representative images are shown. Dashed lines outlined the infarct area. *N* = 10 in each group. ^*^ *p* < 0.05, ^**^ *p* < 0.01,^***^ *p* < 0.001, by Two‐way *ANOVA* (mean ± standard deviation). (F) Neuronal injury in stroke penumbra was evaluated with co‐immunostaining of NeuN (red) and TUNEL (green) at 3d after tMCAO. Representative images of NeuN^+^TUNEL^+^ neurons in stroke are shown. *N* = 4 in each group. ns (not significant), ^*^ *p* < 0.05, ^**^ *p* < 0.01, by Two‐way *ANOVA* (mean ± standard deviation). (G) Infarct volume was quantified with immunostaining of NeuN with coronal sections collected at 14d after tMCAO. Representative images are shown. Dashed lines outlined the infarct area. *N* = 10 in each group. ns (not significant), ^**^ *p* < 0.01,^***^ *p* < 0.001, by Two‐way *ANOVA* (mean ± standard deviation). (H) Representative images of MBP/SMI32 immunostaining and quantification of white matter damage in the corpus callosum (CC), external capsule (EC), striatum (STR), and cortex (CTX) at 14 days post‐tMCAO. *N* = 4 in each group. ^**^ *p* < 0.01, ^***^ *p* < 0.001, by Two‐way *ANOVA* (mean ± standard deviation). (I) Sensorimotor functions of the stroke models were evaluated by adhesive‐removal test, foot‐fault test and rotarod test at 3–14d after tMCAO. *N* = 10 in each group, ^*^ *p* < 0.05, ^**^ *p* < 0.01, ^***^ *p* < 0.01, Tα1 vs. PBS, by Two‐way *ANOVA* (mean ± standard deviation). **^#^** *p* <  0.05, **^##^** *p* < 0.01, **^###^** *P*< 0.001, Tα1 vs. *Ptma^+/−^* +Tα1, by Two‐way *ANOVA* (mean ± standard deviation). (J) Morris water maze test at 10–14 days after tMCAO. Representative images of the swim paths of mice in each group while the platform was present (learning phase) or removed (memory phase). *N* = 10 per group. ^**^ *p* < 0.01, ^***^ *p* < 0.001, by Two‐way *ANOVA* (mean ± standard deviation). ^##^ *p* < 0.01, Tα1 vs. *Ptma* ^+/−^+Tα1, by Two‐way *ANOVA* (mean ± standard deviation).

Given that endogenous Tα1 is essential for neuroprotection, we next investigated whether supplementing the peptide could confer therapeutic benefits. We first validated the brain penetrability of systemically administered Tα1. Tα1 was conjugated with FLAG and administered intraperitoneally (i.p.) Tα1 (300 µg/kg) immediately following tMCAO. We observed an elevated Tα1 levels in both the contralateral (CL, non‐ischemic hemispheres) and ipsilateral (IP, ischemic hemispheres) compared to sham, with the ipsilateral side showing more higher accumulation (Figure 3A). Immunofluorescence of brain sections from FLAG‐Tα1‐injected mice showed FLAG‐Tα1 co‐localized with peri‐infarct neurons (Figure 3B). Then, to exclude antibody cross‐reactivity and confirm peptide integrity, we administered FITC‐conjugated Tα1 and quantified fluorescence in brain homogenates. FITC signal was elevated in both hemispheres post‐tMCAO, with preferential accumulation in the ipsilateral hemispheres (Figure S3A). These data demonstrated that Tα1 crosses the damaged BBB and accumulated at the stroke lesion. Based on this evidence, we administered daily Tα1 treatments to both WT and *Ptma^+/−^* mice following tMCAO. In WT mice, Tα1 treatment led to improved survival rates (Figure 3C) and a rapid, sustained improvement in neurological function (Figure 3D). These functional benefits were underpinned by a dramatic reduction in tissue damage, with smaller infarct volumes in the acute phase (Figure 3E) and notable increase in surviving neurons in the peri‐infarct region (Figure 3F). Besides, Tα1 treatment resulted in less chronic tissue loss (Figure 3G) and mitigation of white matter damage (Figure 3H), which translated into superior long‐term sensory, motor, and cognitive recovery (Figure 3I,J). Notably, Tα1 substantially reversed the exacerbated phenotype in *Ptma* ^+/−^ mice. Compared to the *Ptma^+/−^* vehicle baseline established in Figure 2, Tα1 attenuated neurological deficits and infarct volumes. Furthermore, chronic tissue loss, white matter injury, and functional impairments were similarly ameliorated, although these parameters remained modestly inferior to WT mice receiving Tα1. Intriguingly, this comprehensive neuroprotection was achieved without significant alterations to the post‐stroke neuroinflammatory cytokine profile (Figure S3B–E). This pivotal finding strongly suggested that Tα1's potent therapeutic action in stroke may transcend its classical immune‐centric role, pointing instead toward a direct, non‐canonical protective mechanism on the neurons themselves.

### 3.4 Tα1 Exerts a Direct, Pro‐Survival Effect on Neurons Following Ischemic Insult in Vitro

**FIGURE 4:**
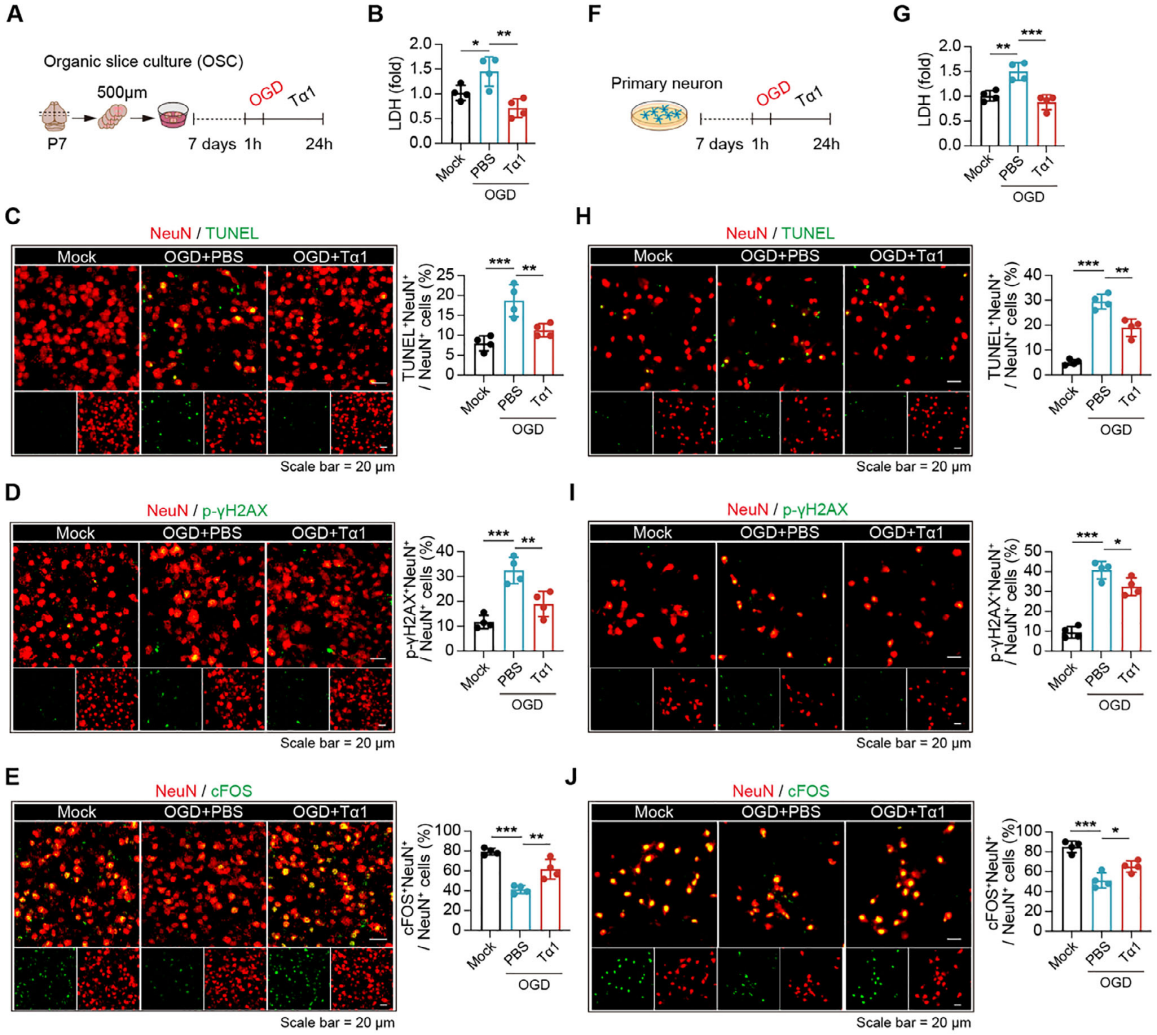
Tα1 exerts a direct, pro‐survival effect on neurons following ischemic insult in vitro. (A–E) Organotypic slice cultures (OSCs) were exposed to 1 h of oxygen‐glucose deprivation (OGD) to mimic ischemic injury and subsequently treated with Tα1 or PBS. Data are representative of 4 biologically independent experiments. (A) Diagram of OSCs experiment. (B) The culture medium was harvested and cytotoxicity was evaluated by measuring LDH release. ^*^ *p* < 0.05, ^**^ *p* < 0.01, compared with PBS by One‐way *ANOVA*. (C) Representative images of NeuN^+^TUNEL^+^ neurons in OSCs are shown. ^**^ *p* < 0.01, ^***^ *p* < 0.001, compared with PBS by One‐way *ANOVA*. (D) Representative images of NeuN^+^p‐γH2AX^+^ neurons in OSCs are shown. ^**^ *p* < 0.01, ^***^ *p* < 0.001, compared with PBS by One‐way *ANOVA*. (E) Representative images of NeuN^+^cFOS^+^ neurons in OSCs are shown. ^**^ *p* < 0.01, ^***^ *p* < 0.001, compared with PBS by One‐way *ANOVA*. (F–J) Primary neuron were exposed to 1 h of OGD to mimic ischemic injury and subsequently treated with Tα1 or PBS. Data are representative of 4 biologically independent experiments. (F) Diagram of primary neuron experiment. (G) The culture medium was harvested and cytotoxicity was evaluated by measuring LDH release. ^**^ *p* < 0.01, ^***^ *p* < 0.001, compared with PBS, by One‐way *ANOVA*. (H) Representative images of NeuN^+^TUNEL^+^ neurons are shown. ^**^ *p* < 0.01, ^***^ *p* < 0.001, compared with PBS by one‐way ANOVA. (I) Representative images of NeuN^+^p‐γH2AX^+^ neurons are shown. ^*^ *p* < 0.05, ^***^ *p* < 0.001, compared with PBS by One‐way *ANOVA*. (J) Representative images of NeuN^+^cFOS^+^ neurons are shown. ^*^ *p* < 0.05, ^***^ *p* < 0.001, compared with PBS, by One‐way ANOVA.

To determine if the neuroprotective effects of Tα1 are mediated by a direct action on neurons, we utilized ex vivo organotypic slice cultures (OSCs) and primary cortical neurons subjected to oxygen‐glucose deprivation (OGD) to mimic ischemic conditions in the absence of systemic factors. Treatment with Tα1 following OGD insult significantly reduced cytotoxicity, as measured by LDH release (Figure 4A,B). Furthermore, Tα1 markedly decreased the number of apoptotic cells and preserved neuronal integrity and activity, as shown by immunofluorescence for TUNEL, p‐γH2AX, and cFOS (Figure 4C–E). These direct neuroprotective effects were consistently replicated in primary cortical neuron cultures (Figure 4F–J) and the neuronal cell line Neuro‐2a (Figure S4A–D). These findings confirm that Tα1's therapeutic benefits are not solely dependent on systemic immune modulation but are mediated by a direct, pro‐survival action on neurons, prompting us to uncover the underlying molecular mechanism.

### 3.5 Tα1 Functions as a Novel Ligand for the Orexin Receptor HCRTR1 on Neurons

**FIGURE 5:**
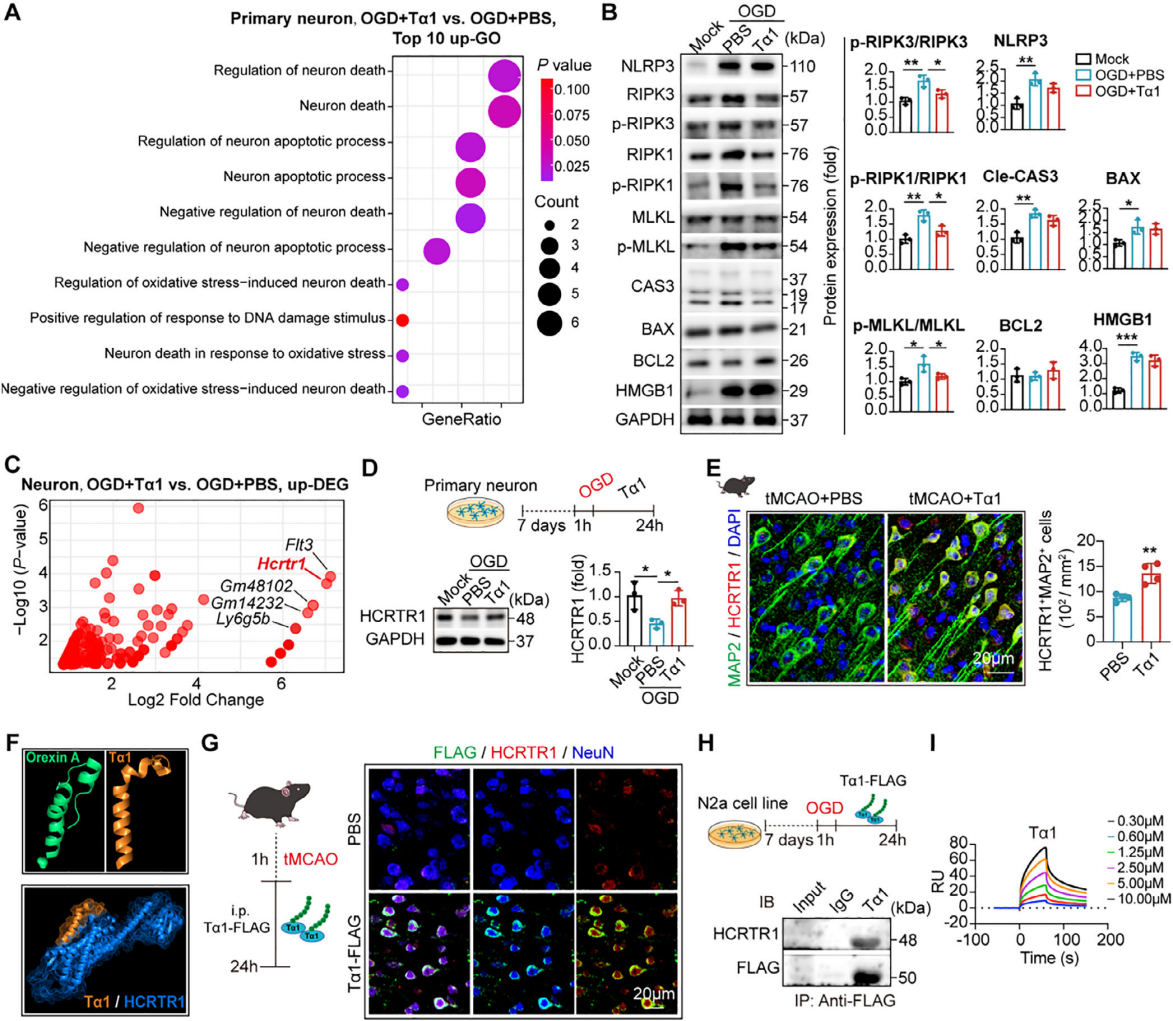
Tα1 functions as a novel ligand for the orexin receptor HCRTR1 on neurons. (A–D) Primary neurons were exposed to 1 h of OGD to mimic ischemic injury and subsequently treated with Tα1 or PBS. Cells were collected for bulk RNA‐sequencing and western blot. (A) Gene Ontology‐biological process (GO‐BP) enrichment of the up‐regulated genes in Tα1 treated neuron. (B) Expression of cell death markers were assessed with Western blot. ^*^ *p* < 0.05, ^**^ *p* < 0.01, compared with PBS by One‐way *ANOVA*. (C) Volcano plot showing the up‐regulated genes in neuron treated with PBS or Tα1. (D) Expression of HCRTR1 in primary neuron was assessed with western blot. ^*^ *p* < 0.05, compared with PBS, by One‐way *ANOVA*. (E) Representative images of MAP2^+^HCRTR1^+^ neurons are shown. N=4 in each group ^**^ *p* < 0.01, compared with PBS by Student's *t* test. (F) Molecular Modeling (up) of Tα1 vs. Orexin A; Molecular docking show the interaction between Tα1 and HCRTR1 (down). (G) Representative images of FLAG^+^HCRTR1^+^NeuN^+^ neurons are shown. (H) Immunoprecipitation (IP) with anti‐FLAG antibodies. IP product was analyzed with immunoblot (IB) to examine the interaction of Tα1 and HCRTR1. (I) Surface Plasmon Resonance (SPR) analyzed the interaction of Tα1 and HCRTR1.

To uncover the molecular pathway governed by Tα1, bulk RNA sequencing was performed on primary neurons subjected to OGD. Tα1 treatment suppressed gene sets related to cell death (Figure 5A). Complementing these findings, we assessed key mediators of necroptosis (RIPK3/RIPK1/MLKL), apoptosis (Bax/BcI2), neuroinflammation (NLRP3), and HMGB1 release, collectively demonstrating the cytoprotective profile conferred by Tα1 (Figure 5B). Unexpectedly, the screen also identified *Hcrtr1*, the gene encoding the Hypocretin (Orexin) Receptor 1, as a significantly upregulated gene following Tα1 treatment (Figure 5C). We validated this key finding at the protein level, observing increased HCRTR1 expression in primary neurons (Figure 5D and Figure S5A) and the Neuro‐2a cell line (Figure S5B,C), as well as in neurons within the peri‐infarct region in vivo (Figure 5E). The identification of HCRTR1, the canonical receptor for the neuropeptide orexin, as a Tα1‐regulated target was highly unexpected. Structural analysis revealed Tα1 exhibits conformational similarity to Orexin A, the endogenous agonist of HCRTR1. This molecular resemblance strongly implies that Tα1 functions as a pharmacological mimetic of Orexin A. Supporting this, molecular docking predicted a high‐affinity interaction between Tα1 and HCRTR1 (Figure 5F). We confirmed this interaction experimentally, showing clear colocalization of FLAG‐Tα1 and HCRTR1 on neurons (Figure 5G). Critically, a direct physical interaction was definitively demonstrated by both co‐immunoprecipitation (Figure 5H) and surface plasmon resonance (SPR) assays (Figure 5I). Together, these data reveal that Tα1 functions as a novel, non‐canonical ligand for HCRTR1, a discovery that led us to test the functional necessity of this interaction for neuroprotection.

### 3.6 The Tα1‐HCRTR1 Axis Protects Neurons by Suppressing RIPK3‐Mediated Necroptosis

**FIGURE 6:**
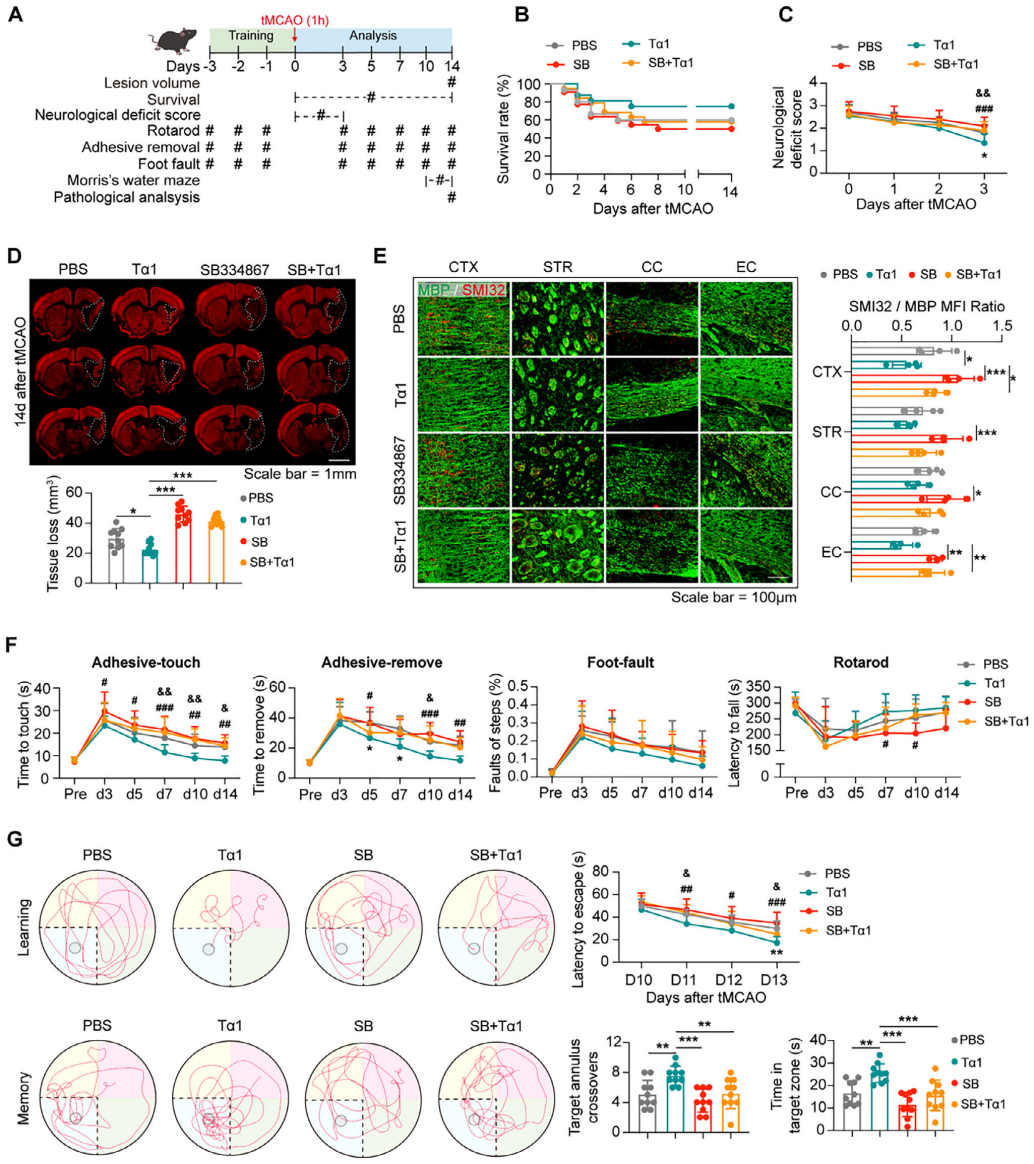
The Tα1‐HCRTR1 axis protects neurons from ischemic injury. (A) Timeline of experimental design. (B) Survival of mice in each group was recorded at 0–14 days after tMCAO. *N* = 15 in PBS and Tα1 group, *N* = 22 in SB group, *N* = 19 in SB+Tα1 group. (C) Neurological deficit score was assessed at 0–3d after tMCAO. *N* = 10 in each group. compared with WT, ^*^ *p* < 0.05, Tα1 group vs. PBS group, by Two‐way *ANOVA* (mean ± standard deviation), **^###^** *P*< 0.001, Tα1 group vs. SB group, by Two‐way *ANOVA* (mean ± standard deviation). **^&&^** *P*< 0.01, Tα1 group vs. SB+Tα1 group, by Two‐way *ANOVA* (mean ± standard deviation). (D) Infarct volume was quantified with immunostaining of NeuN with coronal sections collected at 14d after tMCAO. Representative images were displayed. Dashed lines outlined the infarct area. *N* = 10 in each group. ^*^ *p* < 0.05, ^***^ *p* < 0.001, by Two‐way *ANOVA* (mean ± standard deviation). (E) Representative images and quantification of white matter damage in the corpus callosum (CC), external capsule (EC), striatum (STR), and cortex (CTX) at 14 days post‐tMCAO. *N* = 4 in each group. ^*^ *p* < 0.05, ^**^ *p* < 0.01, compared with WT, by Two‐way *ANOVA* (mean ± standard deviation). (F) Sensorimotor functions of the stroke models were evaluated by adhesive‐removal test, foot‐fault test and rotarod test at 3–14d after tMCAO. *N* = 10 in each group, ^*^ *p* < 0.05, ^**^ *p* < 0.01, Tα1 group vs. PBS group, by Two‐way *ANOVA* (mean ± standard error). **^#^** *p*< 0.05, **^##^** *p*< 0.01, **^###^** *p*< 0.001, Tα1 group vs. SB group, by Two‐way *ANOVA* (mean ± standard deviation). **^&^** *p*< 0.05, **^&&^** *p*< 0.01, Tα1 group vs. SB+Tα1 group, by Two‐way *ANOVA* (mean ± standard deviation). (G) Morris water maze test at 10–14 days after tMCAO. Representative images of the swim paths of mice in each group while the platform were present (learning phase) or removed (memory phase). *N* = 10 per group. ^**^ *p* < 0.01, ^***^ *p* < 0.001. Tα1 group vs. PBS group, by Two‐way *ANOVA* (mean ± standard error). **^#^** *p*< 0.05, **^##^** *p*< 0.01, **^###^** *p*< 0.001, Tα1 group vs. SB group, by Two‐way *ANOVA* (mean ± standard deviation). **^&^** *p*< 0.05, Tα1 group vs. SB+Tα1 group, by Two‐way *ANOVA* (mean ± standard deviation).

To establish whether the neuroprotective effects of Tα1 are mediated specifically through the HCRTR1 receptor, we performed pharmacological intervention experiments in vitro. In neuronal cultures subjected to OGD, pretreatment with the selective HCRTR1 antagonist SB334867 (SB) abolished the protective effects of Tα1 against OGD‐induced cell death. Conversely, treatment with the HCRTR1 agonist Orexin A (OA) phenocopied the pro‐survival effect of Tα1 (Figure S6A–G). These findings demonstrate that Tα1 requires functional HCRTR1 signaling to exert its neuroprotective activity in vitro. Given the in vitro evidence, we next sought to validate this mechanism in vivo. We administered Tα1, SB334867 (SB), or a combination of both following reperfusion (Figure 6A). Consistent with previous observations, Tα1 administration reduced infarct volume and improved neurological deficit scores (Figure 3). Notably, pharmacological blockade of HCRTR1 via SB334867 not only exacerbated ischemic injury when administered alone but also partially attenuated the protective effects conferred by Tα1. While Tα1‐treated mice exhibited robust improvement neurological deficit (Figure 6B,C) and preservation of infarct volume (Figure 6D), white matter damage (Figure 6E) and sensorimotor function (Figure 6F), these benefits were diminished, though not entirely negated, by co‐treatment with SB334867. Additionally, the cognitive enhancement induced by Tα1 was substantially compromised in the presence of SB334867 (Figure 6G), indicating that HCRTR1 activation is integral to Tα1's pro‐cognitive effects. Collectively, these in vivo findings corroborate the in vitro pharmacological evidence, establishing that Tα1‐HCRTR1 interaction is a central axis for mediating neuroprotection and functional recovery after ischemic stroke.

**FIGURE 7:**
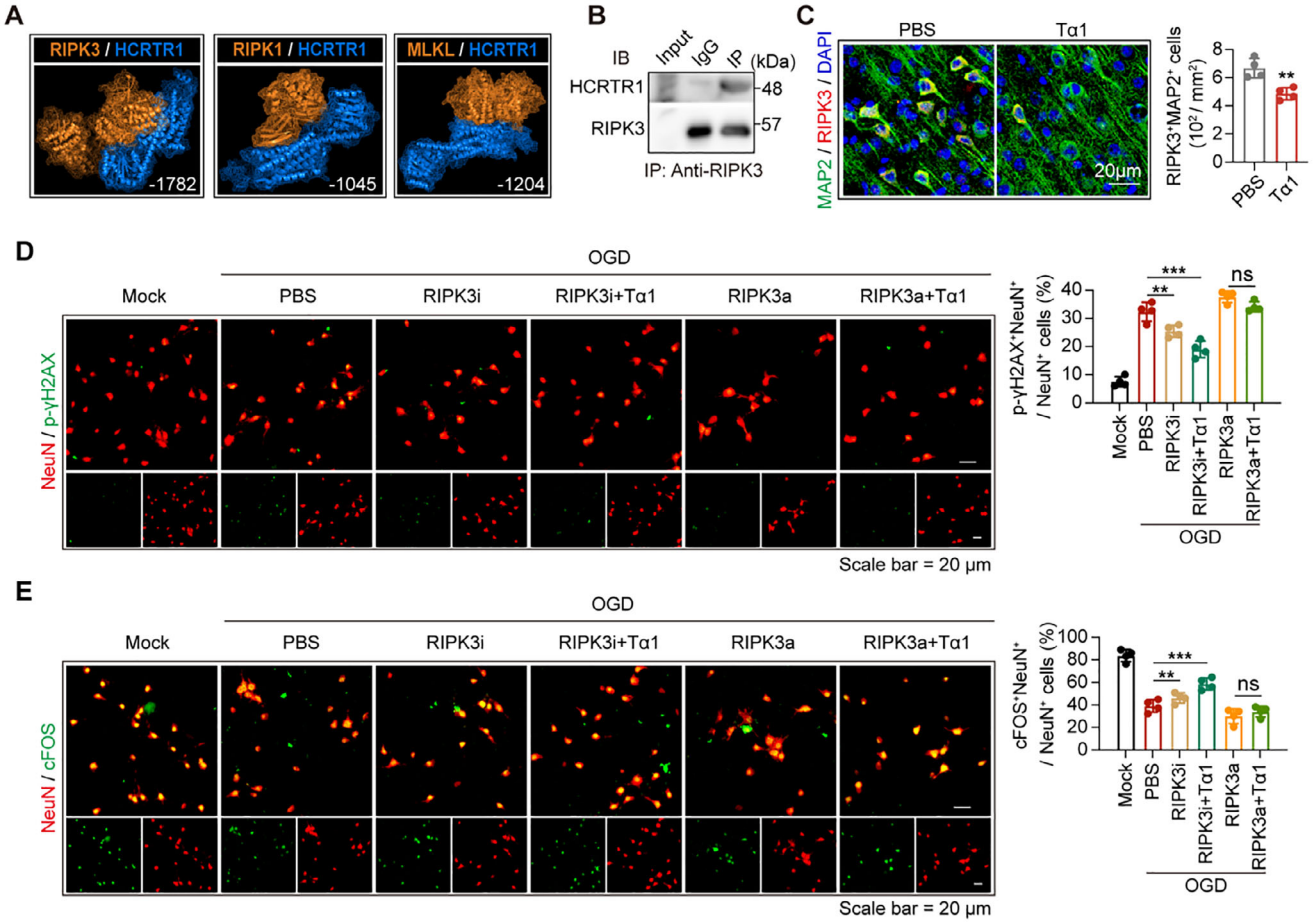
The Tα1‐HCRTR1 axis protects neurons by suppressing RIPK3 mediated necroptosis. (A) Molecular docking shows the interaction between HCRTR1 and RIPK3 (left), HCRTR1 and RIPK1 (middle), and HCRTR1 and MLKL (right), with Model score respectively. (B) Immunoprecipitation (IP) with anti‐RIPK3 antibodies. IP product was analyzed with immunoblot (IB) to examine the interaction of HCRTR1 and RIPK3. Data are representative of three biologically independent experiments. (C) Representative images of MAP2^+^RIPK3^+^ neurons were displayed. *N*=4 in each group. ^**^ *p* < 0.01, compared with PBS by Student's *t* test. (D,E) Primary neuron was isolated and subjected to OGD for 1 h to mimic ischemic conditions, then treated with PBS, RIPK3 inhibitor (RIPK3i), RIPK3 activator (RIPK3a) with or without Tα1. Data are representative of 4 biologically independent experiments. (D) Representative images of NeuN^+^p‐γH2AX^+^ neurons were displayed. ^*^ *p* < 0.05, ^**^ *p* < 0.01, compared with PBS, by Owo‐way *ANOVA*. (E) Representative images of NeuN^+^cFOS^+^ neurons were displayed. ^*^ *p* < 0.05, ^**^ *p* < 0.01, compared with PBS, by One‐way *ANOVA*.

Our initial transcriptomic screen, corroborated by immunoblot analysis of key cell death regulatory proteins, revealed that Tα1 suppresses necroptosis (Figure 5A,B). We therefore investigated the core executioners of this cell death pathway, including Receptor‐Interacting Protein Kinase 3 (RIPK3), RIPK1 and Mixed Lineage Kinase Domain‐Like protein (MLKL) [17]. Molecular docking and co‐immunoprecipitation experiments revealed a novel physical interaction between HCRTR1 and RIPK3 rather than RIPK1 and HCRTR1, suggesting a direct regulatory link (Figure 7A,B). Indeed, Tα1 treatment reduced RIPK3 expression in neurons in the ischemic brain (Figure 7C). Tα1 treatment reduced the levels of phosphorylated RIPK1 (p‐RIPK1), phosphorylated RIPK3 (p‐RIPK3), and phosphorylated MLKL (p‐MLKL) compared to PBS in neuronal cultures in vitro (Figure S7A). Finally, we established the functional hierarchy of this new axis. Pharmacologically activating RIPK3 completely blocked the neuroprotective effects of Tα1. Conversely, inhibiting RIPK3 phenocopied the pro‐survival benefit of Tα1 treatment (Figure 7D,E and Figure S7B–D). Taken together, these results definitively demonstrate that Tα1 protects neurons by activating HCRTR1, which in turn suppresses the downstream effector RIPK3 to inhibit necroptotic cell death.

## 4 Discussion

In this study, we identify a direct, receptor‐mediated signaling pathway bridging the peripheral immune system and the CNS. While Tα1 has shown neuroprotective potential, its mechanism—particularly regarding neuronal death regulation—remained unknown. We demonstrate that the thymus‐derived peptide Tα1 acts as a non‐canonical ligand for the neuronal orexin receptor HCRTR1, suppressing the necroptosis executioner kinase RIPK3 to shield neurons from ischemic injury. These findings redefines Tα1's role, distinguishing its extracellular signaling function from the known intracellular roles of its precursor, Prothymosin alpha (PTMA) and revealing an unexpected function for a canonical neuropeptide receptor.

Traditionally, the thymus's role in neurological disease has been confined to shaping peripheral immunity [18, 19, 20, 21]. Our work challenges this perspective by establishing a direct molecular dialogue between the thymus and neurons. Crucially, Tα1's therapeutic benefits occurred independently of late‐phase neuroinflammatory cytokine profiles, indicating that direct neuroprotection, rather than systemic immunomodulation, is its primary mechanism in the acute phase of injury. This discovery recasts the thymus as an active cerebral defender deploying hormone‐like signals to regulate neuronal fate.

The identification of HCRTR1 as a neuronal Tα1 receptor is unprecedented. While HCRTR1 canonically binds the hypothalamic neuropeptide orexin, a key regulator of arousal, metabolism, and reward [22, 23, 24], our data show that a thymic peptide can co‐opt this receptor. This reveals a surprising promiscuity in GPCR signaling and suggests that endogenous peptides may act on receptors outside their classical pathways.

Mechanistically, our work positions RIPK3‐mediated necroptosis, a major driver of inflammation and cell death in stroke and other neurodegenerative conditions [14, 17, 25, 26, 27, 28, 29], as a key downstream target of the Tα1‐HCRTR1 axis. While RIPK3 inhibitors are being actively explored as therapeutics [30, 31], our study provides a novel, upstream mechanism to control this pathway using an endogenous peptide with a well‐established safety profile. The discovery of a physical interaction between HCRTR1 and RIPK3 suggests a direct regulatory mechanism that warrants further investigation, potentially revealing a new mode of GPCR‐mediated control over cell death machinery. Concomitant with RIPK3 reduction, total RIPK1 and MLKL levels showed modest decreases, likely representing secondary transcriptional feedback rather than direct targeting. These findings carry profound translational implications. Unlike many failed neuroprotective agents that target single pathways [32, 33, 34]. Tα1 presents a uniquely promising candidate by offering a powerful dual‐pronged therapeutic strategy: direct neuronal protection via necroptosis suppression and its known beneficial capacity to mitigate post‐stroke immunosuppression. As an endogenous peptide with a well‐established human safety profile, Tα1 overcomes a major translational hurdle [35, 36, 37].

Our study has some limitations. The use of heterozygous knockout mice, while necessary, provides a conditional, cell‐specific knockout models would further refine our understanding of Tα1's role. Clinical correlations between circulating Tα1 and stroke severity require validation in larger, longitudinal cohorts. Additionally, comprehensive time‐course analyses of immune cell populations would further elucidate Tα1's immunomodulatory effects.

In conclusion, we have identified a novel neuroprotective pathway where the thymic peptide Tα1 engages neuronal HCRTR1 to suppress RIPK3‐mediated necroptosis. This work illuminates a new facet of neuro‐immune biology and nominates the Tα1‐HCRTR1‐RIPK3 axis as a novel, potent, readily translatable target for ischemic stroke and other neurodegenerative disorders.

## Disclosures

The authors have nothing to report.

## Conflicts of Interest

The authors declare no conflicts of interest.

## Data Availabililty Statement

The data that support the findings of this study are available from the corresponding author upon reasonable request.

## Supporting information




**Supporting File**: advs77525‐sup‐0001‐SuppMat.docx.
