# Supplementary material for: Thymosin Alpha‐1 Provides Direct Neuroprotection by Engaging the Orexin Receptor HCRTR1 to Suppress Neuronal Necroptosis
Source: Adv Sci (Weinh). 2026 Sep 3:e22372. Online ahead of print. doi: 10.1002/advs.202522372 (PMC13542392; doi:10.1002/advs.202522372)
Supplement: Supplementary file 1 [file ADVS-9999-e22372-s001.docx]

**Changes are highlighted in yellow.**

**Thymosin alpha-1 provides direct neuroprotection by engaging the orexin receptor HCRTR1 to suppress neuronal necroptosis**

Xinmei Kang^1,2#^, Shisi Wang^1#^, Liubin Zhang^1#^, Mengyan Hu^1^, Xiaotao Su^1^, Yuxin Liu^1^, Qin Qin^1^, Haotong Yi^1^, Liling Yuan^1^, Wei Cai^1^, Lei Wei^1^*, Zhengqi Lu^1,2^*

**Supplementary methods**

**Murine models of acute cerebral ischemic stroke**

Murine models of focal acute ischemic stroke were induced with the procedure of transient middle cerebral artery occlusion (tMCAO) as previously described^1^. Briefly, mice were anesthetized with 1.5–2.0% isoflurane under spontaneous breath. A filament was inserted into the external carotid artery (ECA) and was directed to the middle cerebral artery (MCA) through the internal carotid artery (ICA). Insertion of the filament was maintained for 60 min. Cerebral reperfusion was fulfilled after the filament was retracted. During the surgery, body temperature of mice was monitored and maintained with a heating pad. Cerebral blood flow (CBF) during surgery was measured by laser speckle blood flow imager (SIM BFI HR Pro, SIM Opto-Technology Co., Ltd). Mice with more than 70% reduction of blood flow in the ischemic core were included in the study and mice that died during surgery were excluded. Sham operated animals underwent similar procedure except filament insertion. After surgery, Tα1 (300µg/kg) or PBS was intraperitoneal administration (i.p.) immediately daily for 14 consecutive days.

**Infarct volume measurement**

Infarct volume of stroke mice was evaluated with with NeuN immunostaining, six coronal brain sections with 1 mm interval were stained. The infarct area was determined as the difference between the NeuN^+^ area and NeuN^-^ area in each section and the infarct volume was determined by multiplying the mean area of tissue loss by the distances between the two adjacent brain slices.

**Neurological deficit scoring**

Neurological deficit scores were assessed immediately after ischemia-reperfusion (0 day) and 1–3 d after tMCAO. Neurological deficit scores were determined on a 0–4 scale: 0 = no apparent deficit, 1 = weakness in the ipsilateral forelimb (right), 2 = circulating to the ipsilateral side (right), 3 = 14 W. CAI ET AL.body unbalance and trunk incline to ipsilateral, and 4 = no spontaneous motor activity or death^3^.

**Behavioral tests**

Sensorimotor functions of mice were measured by rotarod test, adhesive&removal test, and foot-fault test at 3–14d after tMCAO.

**Rotarod test.** mice were pre-trained for 3 consecutive days before tMCAO using an accelerated paradigm. Data obtained at the last training test were recorded as a baseline. Tests were performed at 3d, 5d, 7d, 10d and 14d after tMCAO. Mice were placed on a rotating drum with a speed accelerating from 0 to 40 rpm within 5 min and maintained at the final speed. The trial was ended if mouse fell off the drum or gripped the device and spun around for three consecutive revolutions. The latency to fall or spin around on the rung was recorded. Three trials were performed per testing day and the mean values were expressed as data.

**Adhesive touch-removal test.** A 2×3 mm adhesive tape was applied to right fore paws. Tactile responses were measured by recording the time to touch and to remove the adhesive tape. The maximum observation period was 120 s. In each training/testing day, three trials were performed with each mouse. The mean values were recorded as data. Mice were pre-trained for 3 consecutive days before tMCAO and data in the last straining session were set as baseline. Tests were performed at 3d, 5d, 7d, 10d and 14d after tMCAO.

**Foot-fault test.** a stainless-steel grid floor (20 cm × 40 cm with a mesh size of 4cm^2^) was used as the testing field. Each mouse was placed on the grid floor and steps of mice were videotaped for 1 min. The number of total steps and forelimb foot-fault (when the forelimb fell through the grid) was recorded. Mice were pre-trained for 3 consecutive days before tMCAO and data in the last training session were set as baseline. Tests were performed at 3d, 5d, 7d, 10d, and 14d after tMCAO.

**Morris water maze test.** In the learning test, a square platform (11x11 cm^2^ ) was submerged 2 cm beneath the water surface in a circular pool (diameter = 150 cm) of opaque water. Mice were placed into the pool from one of the four locations and allowed to locate the hidden platform for 60 seconds. Each mouse was trained on 3 trials (with randomly assigned starting positions) per day to locate the platform for 3 consecutive days before tMCAO. At the end of each trial, the mouse was placed on the platform or allowed to stay on the platform for 30 seconds with prominent spatial cues displayed around the room. The time spent to reach the platform (learning phase) was recorded. Three trials were performed on each day. The memory test was performed on day 14. The platform was removed, and a single 60 seconds probe trial was conducted. Time spent in the goal quadrant where the platform was previously located was recorded.

**Cell cullture**

Neuro-2a cell line was obtained from American Type Culture Collection (ATCC). The specific identifier for this cell line is RRID: CVCL_0470. This cell line was chosen for its well-established use in neuro-biological research and its documented responsiveness to various neuroprotective agents. To ensure the reliability of our experimental findings, we performed regular mycoplasma testing on the cells. Neuro-2a cells were maintained in DMEM-high glucose medium (Thermo Scientific, C11995500BT) supplemented with 10% fetal bovine serum (FBS) (PAN, P30-3302), and incubated at 37 °C in a humidified atmosphere of 5% CO2.

**Primary neuron culture**

Primary neuron culture was prepared from the whole brains of C57/Bl6 mouse pups (P0) as described previously^4^. Briefly, brain tissue of mouse pups was isolated and transferred to ice-cold HBSS buffer. The meninges were removed the cortex were cut into small pieces. The tissue was then digested in 2.5% trypsin at 37°C for 8 min. The single cells were collected by centrifugation at 300 g for 5 min and seeded with neuron culture media (Neurobasal Medium (Thermo Fisher Scientific), 1% B27 (GIBCO), 0.2μΜ Glutamax (Sigma), and 0.5% P/S). Primary neurons were cultured for 7 days, exposed to OGD for 1 hour, and treated with PBS or Tα1 for 24 hours, followed by subsequent assay.

**Organotypic Slice Cultures**

Organotypic brain slice were prepared from the cerebrum of postnatal day 7 C57/Bl6 mouse pups as described previously^5^. Briefly, cerebrum of mouse pups was isolated and transferred to ice-cold HBSS buffer. The meninges were cleared and the cerebrum were placed on the cutting stage of a McIlwain tissue chopper (CAVEY LAB, MTC-21) and sliced into 500μm thick sections. Then, the organotypic slice were transferred to cell culture insert (pore diameter = 0.4μm) and cultured in 1mL OSCs medium (36.7% Basal medium eagle [BME, Thermo Fisher Scientific], 36.7% Neurobasal-A Medium [Thermo Fisher Scientific], 1% GlutaMAX [GIBCO], 0.033% insulin [Beyotime], and 0.5% P/S, 25% heat-inactivated horse serum [GE Hyclone]). After 24h, the OSCs medium was replaced by fresh medium followed by medium changes every 2 days up to Day 7.

**Western blot**

Protein was extracted with RIPA lysis buffer (Beyotime, P0013J) from the brain or astrocyte. A total amount of 40μg protein of each sample was applied to western blot experiments. Western blot was performed with standard SDS-polyacryamide gel electrophoresis method and HiPer ECL Western HRP Substrate (Mei5bio, MF074). The following primary antibodies were used: rabbit anti-HCRTR1 (Proteintech 18370-1-AP, 1:1000), Necroptosis Antibody Sampler Kit (CST, 98110), rabbit anti-HMGB1 (Proteintech 10829-1-AP, 1:1000), rabbit anti-NLRP3 (Proteintech 30109-1-AP, 1:1000), rabbit anti-CASPASE3 (Proteintech 25128-1-AP, 1:1000), rabbit anti-BAX (Proteintech 50599-2-lg, 1:1000), rabbit anti-BCL2 (Proteintech 12789-1-AP, 1:1000), and mouse anti-GAPDH (Proteintech 60,004-1-Ig, 1:10000), Immunoreactivity was assessed with ImageJ software.

**Immunoprecipitation (IP)**

Protein of N2a cell line was extracted with 1 ml of RIPA lysis buffer (Sigma). Antibodies (2 ug per sample) were applied to the IP system and incubated overnight at 4℃ on a shaker. Pierce protein A/G magnetic beads (Thermo Fisher, Carlsbad, CA, USA, 88802) were then added into the system (10 ll peample). The IP system was then incubated in a rotator for additional 2 hours at 4℃. After three washes with 1 ml RIPA lysis buffer, 100 ll of HCL-Glycine (pH = 2.0) was applied to elute the precipitated protein. The sample was then subjected to Western blot experiments or to liquid chromatography-mass spectrometry (LC-MS) analysis. The following antibodies were used: rabbit (anti-FLAG Proteintech,66008-4-lg), goat anti-RIPK3 (Proteintech, 17563-1-Ap).

**RT-qPCR**

Total RNA from mouse brain tissue was extracted with RNA Quick Purification kit (ESscience, RN001) according to the manufacturer’s instructions. A total of 1μg RNA (OD260nm/280nm =1.8-2.2) was applied to the first strand cDNA synthesis in a 40μl system using Fast Reverse Transcription kit (ESscience, RT001). Real time polymerase chain reaction (RT-qPCR) was performed on a QuantStudio 5 (ABI) quantitative PCR machine using SYBR Green qPCR Mix (GDSBio, P2092a) with 0.5μl of the synthesized cDNA in each reaction with. The following program was performed: 95°C for 30s; 95°C for 5s and 60°C for 34s, repeated for 40 cycles; 95°C for 15s, 60°C for 1min and 95°C for 15s (Melt curve). Primers used in the study are listed in Table S1. Delta CT log2 (compared with the CT value of Actb) was calculated and normalized to the means of control group. Primers were summarized in Table S2.

**Immunofluorenscence**

In *in vivo* experiments, mice were sacrificed at indicated time points. After sufficient perfusion with 20ml of PBS and 20ml of 4% Paraformaldehyde, brains were removed and cut into coronal sections (20μm) on a frozen microtome. Brain sections were washed and incubated with primary antibodies overnight in PBS containing 0.03% Triton-X100 and 3% BSA. After wash, brain sections were incubated with secondary antibodies for 1h at room temperature. The following primary antibodies were used: rabbit anti-NeuN (Abcam ab177487, 1:500), rabbit anti-cFOS (CST 2250, 1:500), rabbit anti-γH2AX (Affinity Biosciences AF3187, 1:500), chicken anti-MAP2 (Abcam ab5392, 1:500), mouse anti-SMI32 (Biolegend, 801702,1:400), rabbit anti-MBP (Proteintech 10458-1-AP, 1:500), mouse anti-FLAG (Biolegend, 801702,1:400), rabbit anti-HCRTR1 (Proteintech 18370-1-AP, 1:1000), rabbit anti-RIPK3 (Proteintech, 17563-1-Ap), The following secondary antibodies were applied: anti-rabbit secondary antibody conjugated with Cy3 (Jackson ImmunoResearch Laboratories 112-545-003, 1:1000), anti-rabbit secondary antibody conjugated with 488 (Jackson ImmunoResearch Laboratories 111-545-003,1:1000), anti-rabbit secondary antibody conjugated with Alexa Fluor 647 (Jackson ImmunoResearch Laboratories 111-605-003, 1:1000), anti-mouse secondary antibody conjugated with Cy3 (Jackson ImmunoResearch Laboratories 115-165-003, 1:1000), anti-mouse secondary antibody conjugated with 488 (Jackson ImmunoResearch Laboratories 115-095-003, 1:1000), anti-mouse secondary antibody conjugated with Alexa Fluor 647 (Jackson ImmunoResearch Laboratories 115-605-003, 1:1000).DAPI Fluoromount-G (Abcam, ab104139) was applied to locate nucleus when indicated. Images were captured with a confocal microscopy (Leica TSC SP8) and processed with ImageJ software by a blinded observer for the unbiased counting of automatically recognized cells and mean fluorescent intensity calculation.

**Enzyme-linked immunosorbent assay (ELISA)**

The concentrations of Tα1,Tβ4 and Tβ10 in human and mice serum or brain tissue were measured using the corresponding ELISA kit (MEIMIAN). ELISA was carried out according to the manufacturer's instructions.

**Supplementary figure**

**Figure S1.**

**Figure S1.**

**(A-D)** Healthy control (HC) (*N* = 15) and AIS patients (*N* = 28) peripheral blood serum and clinical data were collected and subjected to further analysis. **(A)** Tβ4 in peripheral blood were detected by ELISA. Compared to HC, by Student’s *t* test (mean ± standard deviation). **(B)** Tβ10 in peripheral blood were detected by ELISA. Compared to HC, by Student’s *t* test (mean ± standard deviation). **(C-D)** The association between Tα1 with 1d NIHSS **(C),** CRP (C-reactive protein) **(D)** was evaluated with simple linear correlation analysis. **(E)** Tβ4 in mouse peripheral blood were detected by ELISA. Compared to sham; by Students *t* test (mean ± standard deviation). **(F)** Tβ10 in mouse peripheral blood were detected by ELISA. Compared to Sham; by Student’s *t* test (mean ± standard deviation). **(G)** Tβ4 in mouse brain tissue were detected by ELISA. ***P* < 0.01 compared to Sham; by Student’s *t* test (mean ± standard deviation). **(H)** Tβ10 in mouse brain tissue were detected by ELISA. ****P* < 0.001 compared to sham; by Student’s *t* test (mean ± standard deviation).

**Figure S2.**

**Figure S2.**

**(A-C)** Quantitative PCR analysis of inflammatory mediator expression in the ipsilateral ischemic hemisphere at 3d **(A)**, 7d **(B)** and 14d **(C)** after tMCAO. *N* = 3 each group at 3d and 7d, *N* = 4 each group at 14d. **P* < 0.05, ****P* < 0.001 compared with the WT group, by Two-way *ANOVA*.

**Figure S3.**

**Figure S3.**

**(A)** Mice received an immediate i.p. injection of FITC-labeled Ta1 follcwing tMCAO. At 24 hours post-treatment, animals were sacrificed,and brains were collected. Fluorescence intensity was measured in the CL (Contralateral, non-ischemic) and IP (Ipsilateral, ischemic) hemispheres. *N* = 3 in each group.****P*< 0.001, compared with by Sham, by One-way *ANOVA* (mean ± standard deviation). **(B)** Timeline of experimental design. **(C-E)** Quantitative PCR to assess the expression of inflammatory mediators in the ipsilateral ischemic hemisphere at 3d **(C)**, 7d **(D)** and 14d **(E)** after tMCAO. *N* = 3 each group. **P* < 0.05, ***P* < 0.01 compared with the PBS group, by Two-way *ANOVA*.

**Figure S4.**

**Figure S4.**

**(A-D)** Neuro-2a (N2a), a mouse-driven neural cell line were exposed to 1 h of OGD to mimic ischemic injury and subsequently treated with Tα1 or PBS. Data are representative of 4 biologically independent experiments. **(A)** The culture medium was harvested and cytotoxicity was evaluated by measuring LDH release. **P* < 0.05, ***P* < 0.01, compared with PBS, by One-way *ANOVA*. (B) Representative images of NeuN^+^TUNEL^+^ neurons are shown. ***P* < 0.01, ****P* < 0.001, compared with PBS, by One-way *ANOVA*. (C) Representative images of NeuN^+^p-γH2AX^+^ neurons are shown. ***P* < 0.01, ****P* < 0.001,compared with PBS, by One-way *ANOVA*. (D) Representative images of NeuN^+^cFOS^+^ neurons are shown. ***P* < 0.01, ****P* < 0.001,compared with PBS, by One-way *ANOVA*.

**Figure S5.**

**Figure S5.**

**(A)** Primary neuron were exposed to 1 h of OGD to mimic ischemic injury and subsequently treated with Tα1 or PBS. HCRTR1 expression was detected by immunostaining. Representative images show NeuN (green) and HCRTR1 (red) in primary neurons. ***P* < 0.01, ****P* < 0.001, compared with PBS, by One-way *ANOVA*. **(B)** Representative images of NeuN^+^HCRTR1^+^ in N2a cell line were displayed. **P* < 0.05, ****P* < 0.001, compared with PBS, by One-way *ANOVA*. **(C)** HCRTR1 expression in N2a cell line was detected by western blot. **P* < 0.05, ***P* < 0.01, compared with PBS, by One-way *ANOVA*. **(D)** Representative images of NeuN^+^HCRTR1^+^ neurons in OSCs were displayed. **P* < 0.05, ****P* < 0.001, compared with PBS, by One-way *ANOVA.* **(E)** HCRTR1 expression in OSCs was detected by western blot. in OSCs was detected by western blot. **P* < 0.05, compared with PBS, by One-way *ANOVA*.

**Figure S6.**

**Figure S6.**

**(A-D)** Primary neuron were exposed to 1 h of OGD to mimic ischemic injury, then treated with PBS, OrexinA (OA, the well-known HCRTR1 agonist), SB334867 (SB, the well-known HCRTR1 antagonist) and Tα1. Data are representative of 4 biologically independent experiments. **(A)** Representative images of SYTO10 and Ethidium homodimer-2 staining are shown. The number of intact cells (SYTO10^+^ Ethidium homodimer-2^-^) and injured/dead cells (SYTO10^+^ Ethidium homodimer-2^+^) was calculated. **P* < 0.05, ***P* < 0.01, compared with PBS, by One-way *ANOVA*. **(B)** Representative images of NeuN^+^TUNEL^+^ neurons are shown.***P* < 0.01, compared with PBS, by One-way *ANOVA*. **(C)** Representative images of NeuN^+^p-γH2AX^+^ neurons are shown. ****P* < 0.001,compared with PBS, by One-way *ANOVA*. (D) Representative images of NeuN^+^cFOS^+^ neurons are shown. ***P* < 0.01, ****P* < 0.001, compared with PBS, by One-way *ANOVA*. **(E-G)** N2A cell line were exposed to 1 h of OGD to mimic ischemic injury, then treated with PBS, OrexinA, SB334867 and Tα1. Data are representative of 4 biologically independent experiments. **(E)** Representative images of NeuN^+^TUNEL^+^  were displayed. **P* < 0.05, compared with PBS, by One-way *ANOVA*. **(F)** Representative images of NeuN^+^p-γH2AX^+^ neurons were displayed. **P* < 0.05, ***P* < 0.01, compared with PBS, by One-way *ANOVA*. **(G)** Representative images of NeuN^+^cFOS^+^ neurons were displayed.**P* < 0.05, ***P* < 0.01,compared with PBS, by One-way *ANOVA*.

**Figure S7.**

**Figure S7.**

**(A)** N2a cell were exposed to 1 h of OGD to mimic ischemic injury and subsequently treated with Tα1 or PBS. Expression of p-RIPK3/RIPK3, p-RIPK1/RIPK1 and p-MLKL/MLKL was assessed with western blot. Data are representative of 3 biologically independent experiments. **P* < 0.05, ***P* < 0.01, compared with PBS, by One-way *ANOVA*. **(B-D)** N2a cell line were subjected to OGD for 1 hours to mimic ischemic conditions, then treated with PBS, RIPK3i, RIPK3a with or without Tα1. **(B)** Representative images of NeuN^+^TUNEL^+^  were displayed. **P* < 0.05, ****P* < 0.001, compared with PBS, by One-way *ANOVA*. **(C)** Representative images of NeuN^+^p-γH2AX^+^ neurons were displayed. **P* < 0.05, ****P* < 0.001, compared with PBS, by One-way *ANOVA*. **(D)** Representative images of NeuN^+^cFOS^+^ neurons were displayed.**P* < 0.05, ****P* < 0.001, compared with PBS, by One-way *ANOVA*.

**Supplemental tables**

**Table S1.Demographic characteristics of the AIS patients and healthy controls.**

| Demographics | | Healthy control (*N*=15) | | AIS patient (*N*=28) | |
| --- | --- | --- | --- | --- | --- |
| Age, y, median (quartiles) | | 66 (61-74) | | 70 (48-83) | |
| Females sex, *N* (%) | | 4 (27%) | | 5 (18%) | |
| Diabetic mellitus, *N* (%) | | 0 | | 7 (17.1%) | |
| Hypertension, *N* (%) | | 0 | | 19 (46.3%) | |
| Coronary heart disease, *N* (%) | | 0 | | 3 (7.3%) | |
| Infarct volume (cm^3^), median (quartiles) | | / | | 1.66 (0.64-2.69) | |
| WBC (*10^9^/L), median (quartiles) | | / | | 8.69 (5.73-11.6) | |
| CRP (mg/L), median (quartiles) | | / | | 2.65 (1.07-7.01) | |
| 1 NIHSS score, median (quartiles) | | / | | 5 (3-6) | |
| 7 NIHSS score, median (quartiles) | | / | | 2 (1-3) | |
| **Table S2. RT-qPCR primers used in the study.** | | | | |  |
| **Gene** | **Forward primer** | | **Reverse primer** | |  |
| *Il1a* | *ACGGCTGAGTTTCAGTGAGACC* | | *CACTCTGGTAGGTGTAAGGTGC* | |  |
| *Il1b* | *GAAATGCCACCTTTTGACAGTG* | | *TGGATGCTCTCATCAGGACAG* | |  |
| *Il4* | *GGTCTCAACCCCCAGCTAGT* | | *GCCGATGATCTCTCTCAAGTGAT* | |  |
| *Il6* | *CTGCAAGAGACTTCCATCCAG* | | *AGTGGTATAGACAGGTCTGTTGG* | |  |
| *Il9* | *TCCACCGTCAAAATGCAGCTGC* | | *CCGATGGAAAACAGGCAAGAGTC* | |  |
| *Il10* | *CTTACTGACTGGCATGAGGATCA* | | *GCAGCTCTAGGAGCATGTGG* | |  |
| *Il12a* | *CAATCACGCTACCTCCTCTTTT* | | *CAGCAGTGCAGGAATAATGTTTC* | |  |
| *Il17* | *CCTGGACTCTCCACCGCAA* | | *TTCCCTCCGCATTGACACAG* | |  |
| *Il21* | *AATGGCTCCTTCAAAAGATGATTC* | | *TACTCCTGCATTCGTGAGCG* | |  |
| *ccl1* | *GCTTACGGTCTCCAATAGCTGC* | | *GCTTTCTCTACCTTTGTTCAGCC* | |  |
| *ccl9* | *TCCAGAGCAGTCTGAAGGCACA* | | *CCGTGAGTTATAGGACAGGCAG* | |  |
| *cxcl3* | *TGAGACCATCCAGAGCTTGACG* | | *CCTTGGGGGTTGAGGCAAACTT* | |  |
| *cxcl4* | *GTTGTTTCTGCCAGCGGTGGTT* | | *ACAGTGGCGTCCTGCCTTGATC* | |  |
| *cxcl10* | *ATCATCCCTGCGAGCCTATCCT* | | *GACCTTTTTTGGCTAAACGCTTTC* | |  |
| *cxcl11* | *CCGAGTAACGGCTGCGACAAAG* | | *CCTGCATTATGAGGCGAGCTTG* | |  |
| *cxcl12* | *GGAGGATAGATGTGCTCTGGAAC* | | *AGTGAGGATGGAGACCGTGGTG* | |  |
| *Ifna* | *GCCACGGCACAGTCATTGA* | | *TGCTGATGGCCTGATTGTCTT* | |  |
| *Tnfa* | *CTGAACTTCGGGGTGATCGG* | | *GGCTTGTCACTCGAATTTTGAGA* | |  |
| *Arg1* | *CTCCAAGCCAAAGTCCTTAGAG* | | *GGAGCTGTCATTAGGGACATCA* | |  |
| *Mmp8* | *CCACACACAGCTTGCCAATG* | | *GCTTCTCTGCAACCATCGTG* | |  |
| *Mmp9* | *CCAGCCGACTTTTGTGGTCT* | | *TGGCCTTTAGTGTCTGGCTG* | |  |
| *Gapdh* | *CCCTTAAGAGGGATGCTGCC* | | *TACGGCCAAATCCGTTCACA* | |  |

**Reference**

1. FOXP3 macrophage represses acute ischemic stroke-induced neural inflammation. Autophagy 2022; 19: 1144–1163.
2. Neurobehavioral testing in subarachnoid hemorrhage: a review of methods and current findings in rodents. J Cereb Blood Flow Metab 2017; 37: 3461–3474.
3. All trans-retinoic acid protects against acute ischemic stroke by modulating neutrophil functions through STAT1 signaling. J Neuroinflammation. 2019;16(1):175
4. Goshi N, Morgan RK, Lein PJ, Seker E. A primary neural cell culture model to study neuron, astrocyte, and microglia interactions in neuroinflammation. J Neuroinflammation. 2020 May 11; 17(1): 155.
5. Organotypic brain slice cultures: a review. Neuroscience 2015; 305: 86–98
